# Populations in Low-Magnesium Areas Were Associated with Higher Risk of Infection in COVID-19’s Early Transmission: A Nationwide Retrospective Cohort Study in the United States

**DOI:** 10.3390/nu14040909

**Published:** 2022-02-21

**Authors:** Jing Tian, Liwei Tang, Xinwei Liu, Yulan Li, Jinghong Chen, Weiren Huang, Min Liu

**Affiliations:** 1 Shenzhen Key Laboratory of Microbial Genetic Engineering, College of Life Sciences and Oceanography, Shenzhen University, Shenzhen 518060, China; jtian@szu.edu.cn (J.T.); euclase@126.com (L.T.); 2019303006@email.szu.edu.cn (X.L.); 2019301009@email.szu.edu.cn (J.C.); 2 Shenzhen Bay Laboratory, Shenzhen 518055, China; 3 Shenzhen-Hong Kong Institute of Brain Science-Shenzhen Fundamental Research Institutions, Shenzhen 518055, China; 4 Department of Pharmacy, Shenzhen Baoan Center Hospital, Shenzhen 518102, China; lyl0906@yeah.net; 5 International Cancer Center, Health Science Center, Shenzhen University, Shenzhen 518060, China; 6 Shenzhen Institute of Synthetic Biology, Shenzhen Institutes of Advanced Technology, Chinese Academy of Sciences, Shenzhen 518055, China; 7 Department of Urology, Shenzhen Institute of Translational Medicine, The First Affiliated Hospital of Shenzhen University, Shenzhen Second People’s Hospital, Shenzhen 518035, China

**Keywords:** COVID-19, micronutrients, environmental magnesium, magnesium deficiency, health risk

## Abstract

Many studies have confirmed the important roles of nutritional status and micronutrients in the COVID-19 pandemic. Magnesium is a vital essential trace element that is involved in oxidative stress, inflammation, and many other immunological functions and has been shown to be associated with the outcome of COVID-19 infection. Here, we conducted a nationwide retrospective cohort study in the United States involving 1150 counties, 287,326,503 individuals, and 5,401,483 COVID-19 confirmed cases as of 30 September 2020 to reveal the infection risk of the populations distributed in low-magnesium areas in the early transmission of COVID-19. Our results indicate that the average county-level COVID-19 cumulative incidence in low-magnesium areas was significantly higher than in the control areas. Additionally, a significant negative nonlinear association was found between environmental magnesium concentration and the county-level COVID-19 cumulative incidence. Furthermore, the populations distributed in low environmental magnesium areas faced a higher COVID-19 infection risk (RR: 1.066; CI: 1.063–1.068), among which females (RR: 1.07; CI: 1.067–1.073), the 0–17 years subgroup (RR: 1.125; CI: 1.117–1.134), the 65+ years subgroup (RR: 1.093; CI: 1.087–1.098), black people (RR: 1.975; CI: 1.963–1.986), populations outside metro areas, and counties with a smaller population experienced higher risk of infection by COVID-19 than other subgroups. Considering that the magnesium intake of about half the population of the United States is below the daily required dose, our study will contribute to the creation of long-term public health strategies to help protect against COVID-19.

## 1. Introduction

Although vaccination and non-pharmaceutical interventions (NPIs) have played a significant positive role in the containment of coronavirus disease 2019 (COVID-19), the COVID-19 global pandemic is still far from over. Even worse, recent opinions have suggested that COVID-19 could become a long-standing disease causing seasonal epidemics. Thus, understanding the environmental health risk factors associated with COVID-19 and identifying the populations with the greatest COVID-19 burden will contribute to the exploration of low-cost public health policies that may help to prevent or control the spread of COVID-19 in the future.

Numerous studies have revealed the impact of nutritional status and micronutrient intake on the transmission and severity of COVID-19, as well as patient prognosis [1,2,3,4,5,6]. As a vital essential trace element for humans [7,8,9], magnesium is involved in oxidative stress, inflammation, and many other immunological functions [8,10,11] that are involved in infection and infection susceptibility [12,13,14]. Therefore, magnesium is thought to play a role in COVID-19 [11,15,16,17]. According to previous studies, serum magnesium concentration in COVID-19 patients is negatively correlated with the severity of the disease [18,19], i.e., the serum magnesium concentration in patients with severe COVID-19 was significantly lower than that in patients with mild COVID-19 [20]. Additionally, COVID-19 patients with lower serum magnesium concentrations have been shown to have a higher fatality rate [1,2,15]. Other studies focused on older COVID-19 patients also indicating that lower serum magnesium concentrations are associated with poorer outcomes among the elderly [15,21,22]. Magnesium-containing medications have also been used in some cases as supportive care for COVID-19 patients [22,23].

The magnesium contained in the environment affects human health through the drinking water and food that people consume [24,25]. The environmental magnesium concentration can influence the magnesium content in locally sourced water and plants. Around 10% of people’s daily magnesium intake comes from drinking water [26], while the rest comes from food [7,9]. For this reason, previous studies have noted an association between environmental magnesium and some human diseases such as cerebrovascular diseases, ischemic heart diseases, and coronary heart diseases [24,25,27,28]. Considering that magnesium has been reported to be a health factor in COVID-19 patients, it is reasonable to speculate that magnesium in the environment may also play a similar role in COVID-19.

In this paper, we conduct a large retrospective cohort study in the United States using COVID-19 case surveillance data from the US Centers for Disease Control and Prevention (CDC) and county-level environmental magnesium concentration data from the National Geochemical Survey (NGS) to investigate the health risk of populations in low-magnesium areas during the COVID-19 pandemic. In doing so, we reveal the corresponding infectious risk of different population subgroups (gender, age, race, region, and population size) in low-magnesium areas of the United States during the early transmission stage of the COVID-19 pandemic. As nearly half the population of the United States has some degree of magnesium deficiency [29], our study will help to provide both new insights into the role of micronutrients in COVID-19 transmission and evidence to assess the effectiveness of magnesium supplementation as a long-term health intervention in magnesium-deficient populations. This will facilitate long-term COVID-19 prevention and control, especially in the event that COVID-19 becomes a seasonal epidemic.

## 2. Methods

### 2.1. Data and Study Population

In this retrospective cohort study, we used COVID-19 case surveillance data from the US CDC and county-level environmental magnesium concentration data from the US NGS. The period of confirmed COVID-19 cases we covered was from 1 January 2020 to 30 September 2020. A total of 287,326,503 individuals and 5,401,483 confirmed COVID-19 cases were included.

### 2.2. Measures of Variables

The variables in our study included the individual characteristics of confirmed COVID-19 patients (gender, age, and race), the county-level demographic characteristics (population, Rural-Urban Continuum Codes, and the population percentile), and the county-level environmental magnesium concentration. Among these variables, the exposure variable was the environmental magnesium concentration and the outcome variables were the COVID-19 confirmed cases and the percentage of COVID-19 cumulative incidence as of 30 September 2020. In the subgroup analysis, the COVID-19 confirmed cases with missing variables were excluded from the corresponding subgroups. There were 26,031 (0.482%), 6156 (0.114%), and 1,400,833 (25.934%) individuals with missing variables in terms of gender, age, and race, respectively. 

### 2.3. Statistical Analysis

The counties involved in this study were divided into two cohorts based on their environmental magnesium concentration. The low environmental magnesium group included the 25% of counties with the lowest environmental magnesium concentrations (the lower quartile, *n* = 378), while the other counties were included in the control group (*n* = 1132). The statistical significance of mean comparison was conducted using Student’s *t*-test. Two-sided *p*-values of less than 0.05 were considered statistically significant in this study.

The generalized additive model (GAM) was used to estimate the descriptive nonlinear exposure–response nonlinear association between environmental magnesium concentration and cumulative COVID-19 cumulative. Natural cubic spline functions with three to seven degrees of freedom (df) were selected to establish the exposure–response nonlinear association in the GAM analysis.

The subgroup analyses were conducted based on the individual characteristics and residential location characteristics of confirmed COVID-19 cases. The gender (male, female), age (0–17 years, 18–49 years, 50–64 years, 65+ years), and race (white, black, and other) of COVID-19 infected individuals, the RUCC (1, 2, 3; 4, 6, 8; and 5, 7, 9), and the population percentile (Quartile 1 to 4) of counties were included in our subgroup analyses. The relative risk (RR) and the corresponding 95% confidence intervals (CIs) were performed using the Chi-square test.

### 2.4. Sensitivity Analysis

We adopted various methods to test the robustness of our main results. First, we assessed the nonlinear exposure–response association between the environmental magnesium concentration and the COVID-19 cumulative incidence under different degrees of freedom in a generalized additive model. Second, a stratified analysis was conducted to compare the potential differences between the subgroups. Third, we calculated the population-attributable fraction of the population distributed in low-magnesium areas to estimate the infectious attribution of COVID-19 under low magnesium level conditions.

All data processing and analysis was performed using R version 3.6.2.

## 3. Results

### 3.1. The Baseline Characteristics of the COVID-19 Cumulative Incidence and the Environmental Magnesium Concentrations

Our study included the cumulative incidence of COVID-19 as of 30 September 2020 in 1510 counties in the United States. This time period was the first wave of the COVID-19 pandemic and can be used to represent the early stages of transmission of COVID-19 in the United States. The baseline characteristics of the COVID-19 cumulative incidence are listed in Table 1. The total cumulative incidence in the 1510 counties in the United States was in the range of 0.000839% to 9.265% with a mean value of 1.772% and a median value of 1.574%. Significant differences were observed in terms of cumulative incidence between different age and race subgroups. In contrast, no significant differences were observed in terms of cumulative incidence between gender, regional position, and population size subgroups.

The descriptive statistics of the county-level average environmental magnesium concentrations in the United States are shown in Table 2. The low-magnesium group included the 25% of counties with the lowest environmental magnesium concentrations (the lower quartile, *n* = 378), while the other 1132 counties were included in the control group. The mean environmental magnesium concentrations in all areas, low-magnesium areas, and control areas were 0.64, 0.111, and 0.816 weight percent (wt%) and the median of those three groups were 0.496, 0.106, and 0.638 wt%, respectively.

**Figure 1:**
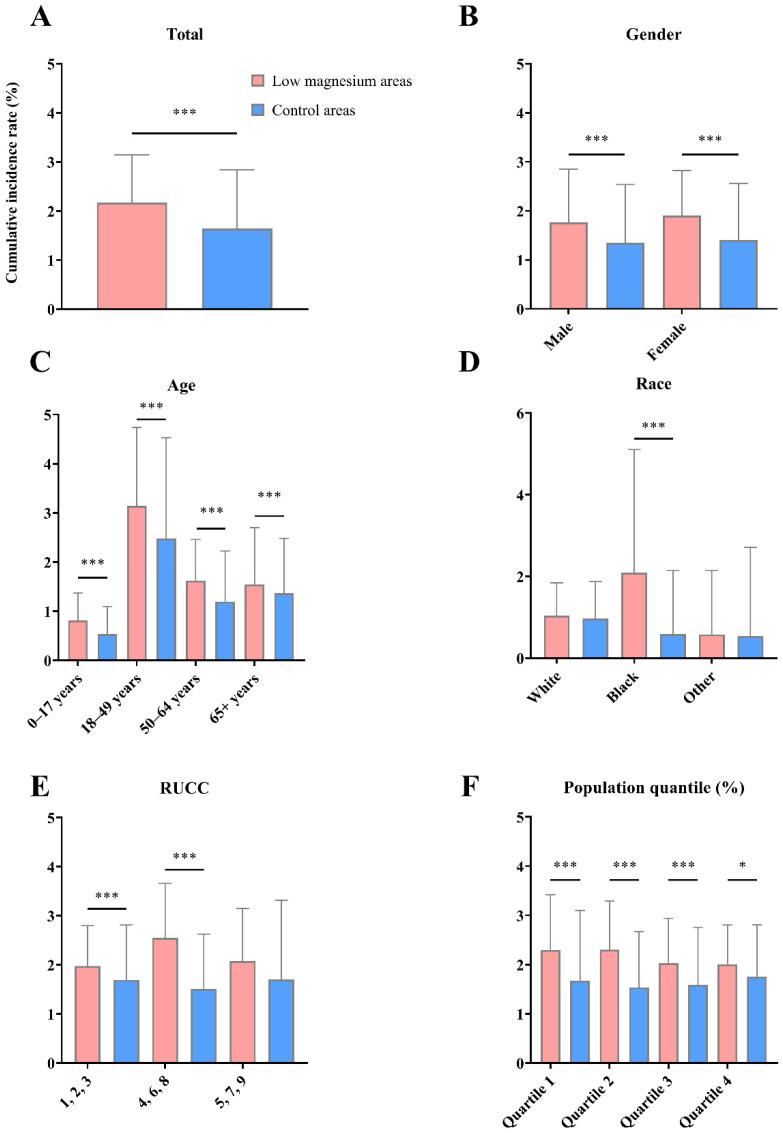
The county-level COVID-19 cumulative incidence in low-magnesium areas and control areas as of 30 September 2020 in the United States. (**A**). The cumulative incidence in the whole population. (**B**–**F**). The subgroup analysis between gender, age, race, RUCC, and county population size groups, respectively. The statistical significance of mean comparison was calculated using Student’s *t*-test. Significance: * for *p* < 0.05, *** for *p* < 0.001.

Then, we compared the county-level COVID-19 cumulative incidence between the low-magnesium areas and the control areas. The results are shown in Figure 1. The average county-level COVID-19 cumulative incidence in low-magnesium areas was significantly higher than in control areas, with the mean values being 2.165% and 1.641%, respectively. In terms of gender, age, and population size, similar significant differences were also found in all subgroups. In addition, the average county-level COVID-19 cumulative incidence in the black race subgroup and Rural-Urban Continuum Codes (RUCC) 1,2,3 (counties located in a metro area) and 4,6,8 (counties adjacent to a metro area) in the regional position group were also significantly higher in the low-magnesium areas compared to the control areas.

**Figure 2:**
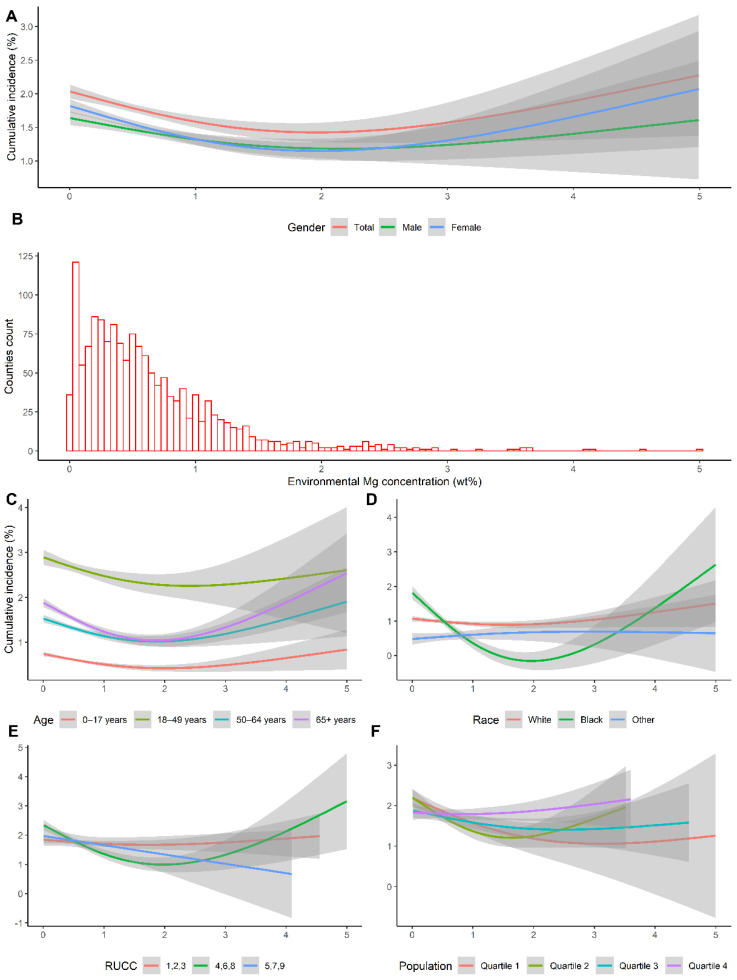
The exposure–response curves of the county-level environmental magnesium concentration and the corresponding COVID-19 cumulative incidence. The x axis represents the county-level environmental magnesium concentration. The y axis indicates the contribution of the smoother to the COVID-19 cumulative incidence. The shaded areas are the 95% confidence intervals. (**A**). The exposure–response curves of the total COVID-19 cumulative incidence and gender subgroup cumulative incidence. (**B**). The distribution of the county-level environmental magnesium concentration. (**C**–**F**). The exposure–response curves of the age, race, RUCC, and population subgroup cumulative incidence, respectively.

Next, we explored the nonlinear association between the environmental magnesium concentrations and the county-level COVID-19 cumulative incidence. The exposure–response curves are shown in Figure 2. The exposure–response curves indicated that significant monotonic nonlinear negative associations existed between environmental magnesium concentrations and the county-level COVID-19 cumulative incidence in all subgroups with narrow confidence intervals when the environmental magnesium concentration was under 2 wt%. Additionally, environmental magnesium concentrations in more than 96% of counties were under 2 wt% (1454 counties and 56 counties were below and above 2 wt%, respectively). This generally echoed the results of the mean comparison between these two groups.

### 3.2. Baseline Characteristics and the COVID-19 Infectious Risk in the Cohorts of Low-Magnesium Areas and Control Areas

Our study included all confirmed COVID-19 cases as of 30 September 2020 in 1510 counties in the United States. A total of 287,326,503 individuals and 5,401,483 COVID-19 confirmed cases were included in our nationwide retrospective cohort study during the period of 1 January 2020 to 30 September 2020. The distribution of each population subgroup and the corresponding confirmed COVID-19 cases in the low-magnesium group and control group are shown in Table 3. The total population of the low-magnesium areas and the control areas were 56,338,459 and 230,988,044, respectively, while the number of confirmed COVID-19 cases in these areas were 1,114,254 and 4,287,229, respectively. Low environmental magnesium levels were significantly associated with the incidence rate of COVID-19 among all groups according to the Chi-square test.

We further assessed the relative risk (RR) and the population attributable fraction (PAF) of the COVID-19 infection risk of the population in low-magnesium areas. The results are shown in Table 4. Compared to the population in control areas, the population in low-magnesium areas experienced a 6.6% (RR: 1.066; CI: 1.063–1.068) higher risk of COVID-19 infection, while the contribution fraction was 1.27% (CI: 1.228–1.312). In the subgroup analysis, female individuals (RR: 1.07; CI: 1.067–1.073), the 0–17 years age subgroup (RR: 1.125; CI: 1.117–1.134), the 65+ years age subgroup (RR: 1.093; CI: 1.087–1.098), black people (RR: 1.975; CI: 1.963–1.986), populations outside metro areas, and counties with smaller populations experienced significantly higher COVID-19 infection risks than the other subgroups. Additionally, the results of the population attributable fraction indicated similar characteristics among these subgroups.

## 4. Discussion and Conclusions

Our study aimed to estimate the COVID-19 infection risk of populations in low-magnesium areas in the United States by conducting a nationwide retrospective cohort study that included 1150 counties, 287,326,503 individuals, and 5,401,483 confirmed COVID-19 cases. The principal findings of this study were as follows. First, the COVID-19 cumulative incidence as of 30 September 2020 in the counties located in low-magnesium areas was significantly higher than in other counties. Second, a significant negative association was found between COVID-19 cumulative incidence and environmental magnesium concentration. Third, populations in low-magnesium areas faced a higher COVID-19 infection risk than populations in other areas, particularly in suburban areas, rural areas, and counties with a small population. Last, the differences in COVID-19 infection risk between gender, age, and race groups were studied and we found that black people, females, and individuals below 18 years or above 65 years of age were associated with higher infection risk in low-magnesium areas compared to the other subgroups.

**Figure 3:**
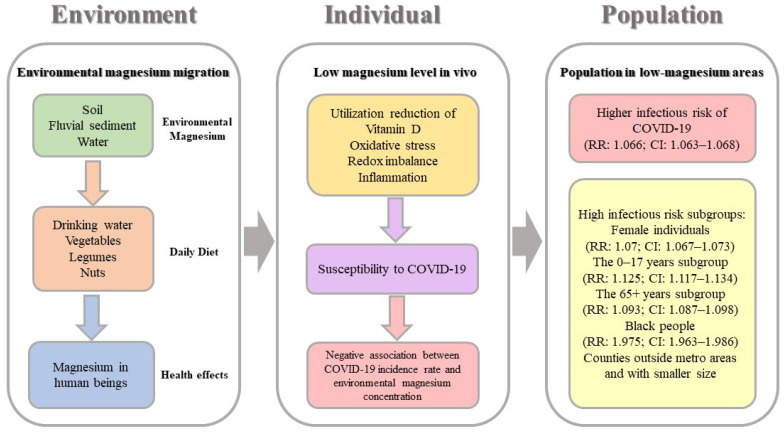
The possible mechanisms of how environmental magnesium affects COVID-19 susceptibility.

The magnesium in the environment is able to affect human health through foods and water [25,27,30,31]. As the magnesium concentration of the soil can influence the magnesium content in plants, especially when large amounts of potash fertilizer are used [32,33,34], the population living in low-magnesium areas are faced with lower magnesium concentrations in their daily drinking water and foods. Considering many studies have indicated the potential impact of magnesium in the environment and in daily diets to many human diseases [24,28,35,36,37,38], it is reasonable to speculate that environmental magnesium might play a similar role in COVID-19 and our findings demonstrate this possibility (as summarized in Figure 3).

Since the beginning of the COVID-19 pandemic, the important role of magnesium in the prevention and treatment of COVID-19 has been noted by researchers [3,18,19,22,39]. However, the risks posed by COVID-19 to the magnesium-deficient population require further study. Our results provide some evidence to suggest that the magnesium-deficient population in the United States has a higher risk of COVID-19 infection.

The possible mechanisms behind the association between magnesium deficiency and the risk of COVID-19 infection that was observed in our study may be related to the immunoregulatory effects of magnesium and its complex activation relationship with vitamin D3. The immunoregulatory effects of magnesium have been proven to involve in variety of human diseases by some previous experimental and clinical studies. Accordingly, magnesium has been shown to have a potential protective effect on type 2 diabetes (T2D)-associated cardiovascular risk in both diabetic and nondiabetic individuals [40]. Magnesium supplementation can have beneficial effects on erythrocyte homeostasis by both reducing the disruption of band 3 protein and maintaining intracellular GSH levels under oxidative stress conditions [41]. Furthermore, as a main kind of N-methyl-d-aspartate (NMDA) receptor antagonist, magnesium is involved in the prophylaxis and treatment of pain by blocking calcium channels, preventing the development of central sensitization, and abolishing established hypersensitivity [42]. Similarly, oxidative stress and redox imbalance are the hallmarks of COVID-19 infection [43,44,45]. An increase in TNF-α, inflammatory interleukins, and mitochondrial ROS has been observed in COVID-19 patients and this process is involved throughout COVID-19 infection and prognosis [46,47,48]. Thus, higher levels of oxidative stress, redox imbalance, and chronic inflammation in the magnesium-deficient population may cause higher infection risk and poorer outcomes when it comes to COVID-19. On the other hand, magnesium, as the cofactor of vitamin D metabolism, is able to improve the utilization of vitamin D [49,50] while low vitamin D levels in vivo are considered to be a risk factor for COVID-19 infection [51,52,53]. Furthermore, the other immune functions of magnesium such as acquired immune responses, homeostasis, and activation of immune cells may also affect individuals’ susceptibility to COVID-19 infection [14,54,55]. Nevertheless, as the complex mechanisms between magnesium and COVID-19 infection are still poorly understood, more studies in this field are needed in the future.

Some implications can be drawn from this study. First, as previous studies have indicated, the magnesium intake of almost half the population of the United States is under the daily required dose. Thus, more attention should be paid to the increased COVID-19 infection risk among this segment of the population, especially when considering that similar conditions may prevail in other countries. Second, different infection risks were observed between different subgroups. Females, black people, individuals aged under 18 years or above 65 years, and population outside metro areas face higher infection risks according to our study. A previous survey on magnesium intake revealed a higher rate of magnesium intake deficiency among children, the elderly, females, and black people in the United States, which is consistent with our observations. The reason for such phenomena may be related to the economic situation and living habits of these populations. For instance, more vegetables and less sugar in one’s daily diet will provide more magnesium intake from food. Eating food from a wider range of sources and drinking mineral water may also increase one’s magnesium intake. Studies on these populations and the health benefits of magnesium supplementation during the COVID-19 pandemic will likely be of great practical significance. Third, the global COVID-19 pandemic is ongoing and is likely to become a long-standing infectious seasonal epidemic disease similar to other common human coronavirus diseases. Our findings may contribute to future public health strategies that may change this situation. When facing a seasonal epidemic, the social and economic costs should be considered. Micronutrient supplementation among specific populations may become an effective public health strategy for dealing with seasonal COVID-19 epidemics that comes with a low social cost.

There were several limitations to this study. First, an analysis of the daily magnesium intake in different counties was not included in our study. The reason for this limitation was that these data were unavailable. Second, some of the racial statistics surrounding COVID-19 infection used in this study were missing. These missing data may affect the accuracy of the subgroup analysis in terms of race. Although we used several methods to improve the robustness of our results, we did not include racial subgroups other than white or black, such as American Indian, Asian, Pacific Islander, and multiple races. This was because the proportion of confirmed COVID-19 cases in these other subgroups was very small during the early stages of COVID-19 transmission. Lastly, economic status and living habits should also be considered in the subgroup analysis. We tried our best to obtain data on these factors but the relevant data were not available. We will continue to focus on this research area, and these factors will be considered in a future study once these data become available.

In conclusion, this study revealed the increased risk of COVID-19 infection among populations living in areas with low environmental magnesium levels in the United States by conducting a nationwide retrospective cohort study. The findings of our study provide some new insights into the complex effects of micronutrients on human health and will contribute to the long-term prevention of COVID-19 in the future.

## Figures and Tables

**Table 1:** Baseline characteristics of the COVID-19 cumulative incidence as of 30 September 2020 in the 1510 counties in the United States.

Variables ^a^	Mean	SD	Min	P25	P50	P75	Max	*p*-Value ^b^
Total	1.772	1.17	0.000839	0.951	1.574	2.303	9.265	
Gender								0.0579
Male	1.451	1.18	0	0.616	1.234	1.971	8.172	
Female	1.531	1.119	0	0.694	1.386	2.123	8.076	
Age								<0.0001
0–17 years	0.601	0.579	0	0.17	0.45	0.887	3.632	
18–49 years	2.646	1.965	0	1.269	2.286	3.5	17.611	
50–64 years	1.302	1	0	0.609	1.129	1.727	7.510	
65+ years	1.51	1.134	0	0.693	1.269	2.132	7.797	
Race								<0.0001
White	0.984	0.881	0	0.372	0.76	1.341	7.151	
Black	0.971	2.128	0	0	0	0.975	21.809	
Other	0.553	2.031	0	0	0	0.41	48.663	
RUCC								0.966
1,2,3	1.765	1.056	0.000839	1.051	1.587	2.241	8.190	
4,6,8	1.779	1.201	0.0318	0.867	1.628	2.456	6.256	
5,7,9	1.783	1.519	0.0193	0.665	1.466	2.43	9.265	
Population								0.1944
Quartile 1	1.857	1.373	0.0318	0.888	1.535	2.541	9.265	
Quartile 2	1.713	1.148	0.0546	0.867	1.528	2.25	6.256	
Quartile 3	1.704	1.122	0.000839	0.947	1.511	2.133	8.190	
Quartile 4	1.813	1.003	0.0367	1.12	1.688	2.349	6.622	

^a^ The COVID-19 cumulative incidence is shown in percent (%). ^b^ The *p*-values were calculated using the one-way ANOVA test.

**Table 2:** Descriptive statistics of environmental magnesium concentrations in the low-magnesium areas and control areas.

	Counties Count	Mean	SD	Min	P25	P50	P75	Max
All areas	1510	0.64	0.599	0.005	0.231	0.496	0.871	4.995
Low-magnesium areas	378	0.111	0.0717	0.005	0.0401	0.106	0.177	0.231
Control areas	1132	0.816	0.593	0.231	0.421	0.638	1.015	4.995

Environmental magnesium concentration is given by wt%.

**Table 3:** Baseline characteristics of the confirmed COVID-19 patients in different groups.

Variables	Low-Magnesium Areas		Control Areas		*p*-Value ^a^
	COVID-19 Cases	Population	COVID-19 Cases	Population	
Total	1,114,254	56,338,459	4,287,229	230,988,044	<0.01
Gender					
Male	489,331	27,520,526	1,960,303	112,780,099	<0.01
Female	555,367	28,609,448	2,127,123	117,242,538	<0.01
Age					
0–17 years	92,620	12,788,830	337,425	52,434,286	<0.01
18–49 years	609,514	20,056,491	2,409,001	82,231,744	<0.01
50–64 years	219,764	14,309,969	852,002	58,670,963	<0.01
65+ years	163,735	9,183,169	614,444	37,651,051	<0.01
Race					
White	431,091	42,719,296	1,560,526	175,149,390	<0.01
Black	173,282	7,634,778	359,800	31,302,641	<0.01
Other	53,822	5,984,384	294,488	24,536,014	<0.01
RUCC					
1,2,3	952,050	49,644,619	3,945,039	208,483,711	<0.01
4,6,8	131,092	5,123,285	240,718	16,317,202	<0.01
5,7,9	30,195	1,517,173	102,389	6,240,513	<0.01
Population					
Quartile 1	68,193	2,930,828	119,727	7,213,034	<0.01
Quartile 2	93,784	4,054,703	207,971	13,603,546	<0.01
Quartile 3	198,560	9,942,781	456,803	28,670,078	<0.01
Quartile 4	752,800	39,356,765	3,503,645	181,554,768	<0.01

^a^ The *p*-values were calculated using the Chi-square test.

**Table 4:** The relative risk (RR) and population attributable fraction (PAF) of the COVID-19 infection risk of the population distributed in low-magnesium areas.

Variables	RR	95% CI	PAF (%)	95% CI
Total	1.066	1.063–1.068	1.27	1.228–1.312
Gender				
Male	1.023	1.02–1.026	0.448	0.387–0.51
Female	1.07	1.067–1.073	1.354	1.294–1.413
Age				
0–17 years	1.125	1.117–1.134	2.4	2.248–2.553
18–49 years	1.037	1.034–1.04	0.727	0.672–0.783
50–64 years	1.058	1.053–1.062	1.116	1.022–1.21
65+ years	1.093	1.087–1.098	1.782	1.671–1.894
Race				
White	1.133	1.129–1.136	2.534	2.464–2.605
Black	1.975	1.963–1.986	16.044	15.889–16.199
Other	0.753	0.746–0.760	−5.079	−5.222–−4.920
RUCC				
1,2,3	1.013	1.011–1.016	0.258	0.215–0.301
4,6,8	1.734	1.723–1.746	14.93	14.731–15.13
5,7,9	1.213	1.198–1.229	3.999	3.722–4.278
Population				
Quartile 1	1.402	1.389–1.415	10.401	10.099–10.704
Quartile 2	1.513	1.501–1.525	10.537	10.325–10.749
Quartile 3	1.253	1.247–1.26	6.125	5.977–6.274
Quartile 4	0.991	0.989–0.994	−0.158	−0.201–−0.114

## Data Availability

The datasets used in this study are all open source and can be found online at the following websites. The COVID-19 case surveillance dataset: https://data.cdc.gov/Case-Surveillance/COVID-19-Case-Surveillance-Public-Use-Data-with-Ge/n8mc-b4w4, accessed date: 14 February 2022. The county-level environmental magnesium concentration dataset: https://mrdata.usgs.gov/geochem/doc/averages/countydata.htm, accessed date: 14 February 2022.
